# The central mechanotransducer in osteoporosis pathogenesis and therapy

**DOI:** 10.3389/fendo.2025.1658967

**Published:** 2025-09-23

**Authors:** Chaoyue Liu, Jihao Yang, Zengsheng Dong, Shuqing Zhao, Zeng-Hui Tian, Ying-Ying Li, Yan-Ke Hao, Mingliang Wang

**Affiliations:** ^1^ Shandong University of Traditional Chinese Medicine, Jinan, China; ^2^ Guizhou University of Traditional Chinese Medicine, Guiyang, China; ^3^ Rizhao Hospital of Traditional Chinese Medicine, Rizhao, China; ^4^ Shanghai University of Traditional, Chinese Medicine, Shanghai, China; ^5^ Shandong University of Traditional, Chinese Medicine Affiliated Hospital, Jinan, China

**Keywords:** Piezo1, osteoporosis, mechanotransduction, bone homeostasis, therapeutic target

## Abstract

This review identifies the mechanosensitive ion channel Piezo1 as the central regulator of bone homeostasis. Piezo1 senses mechanical loads in osteocytes, osteoblasts, and bone marrow mesenchymal stem cells (BMSCs), converting them into Ca^2+^-dependent signals that activate key pathways, including CaMKII, YAP/TAZ, Wnt/β-catenin, and ERK. These cascades collectively promote osteoblast differentiation and suppress osteoclastogenesis via OPG/RANKL modulation. Age-related Piezo1 decline impairs bone mechanoresponsiveness, driving both senile and disuse osteoporosis. Piezo1 also integrates bone metabolism with vascular–immune interactions (e.g., promoting VEGFA release from bone marrow macrophages via the CaN/NFAT/HIF-1α pathway) and the gut–bone axis (e.g., intestinal Piezo1 deletion relieves osteoblast proliferation inhibition by reducing serotonin levels). Therapeutically, Piezo1 agonists restore bone mass in osteoporosis models by reactivating mechanotransduction, while physical interventions achieve similar effects. Outstanding challenges include optimizing mechanical parameters (e.g., vibration frequency, ultrasound intensity) for individualized therapy, disentangling pathway crosstalk under aging and inflammation, and developing bone-targeted delivery systems for Piezo1 modulators. Overall, Piezo1 emerges as a pivotal therapeutic target for osteoporosis.

## 1 Introduction

Osteoporosis (OP) is characterized by reduced bone mass and deteriorated microarchitecture, leading to increased fragility and fracture risk (1, 2). Its pathology arises from an imbalance in bone remodeling: excessive osteoclast-mediated resorption coupled with insufficient osteoblast-driven formation (3, 4). Mechanical stress is a fundamental determinant of skeletal remodeling—loading (e.g., exercise) enhances bone mass and strength, while unloading (e.g., bed rest, spaceflight) induces rapid bone loss (1, 5).

The mechanosensitive ion channel Piezo1 shows age-related decline, which correlates with impaired skeletal responsiveness to mechanical cues and contributes to bone loss in aging (6). In pathological models, the Piezo1 agonist Yoda1 restores mechanotransduction, improving bone mass and strength in glucocorticoid-induced and disuse osteoporosis (7).

Current studies mostly focus on Piezo1’s local role in bone cells, while its systemic regulatory mechanisms in the gut-bone axis and vascular-immune axis have not been systematically summarized; existing Piezo1 agonists (e.g., Yoda1) have off-target risks and long-term safety controversies, and the clinical translation path remains unclear—this review aims to address these gaps by integrating Piezo1’s local and systemic functions in osteoporosis.

Piezo1 is broadly expressed in osteocytes, chondrocytes, and BMSCs. By converting mechanical forces such as fluid shear stress (FSS) into cellular signals, Piezo1 regulates diverse processes including skeletal development, angiogenesis, and immune responses (8). In osteocytes and chondrocytes, Piezo1 modulates osteogenesis and cartilage homeostasis (9); in BMSCs, it promotes osteogenic differentiation while inhibiting adipogenesis (10). Importantly, Piezo1-mediated FSS suppresses osteoporosis progression by reducing RANKL secretion from osteocytes (11). In ovariectomized animal models, Piezo1 also exhibits anti-osteoporotic effects. Genetic studies show Piezo1 polymorphisms (e.g., rs4238686, rs11643303) are associated with human OP: the rs4238686 variant reduces Piezo1 channel opening efficiency, leading to decreased mechanosensitivity of osteocytes and significant correlation with reduced bone mineral density (BMD) in elderly women (12); additionally, Piezo1 expression is markedly reduced in patient bone tissue (9, 12, 13).

### 1.1 Piezo1-mediated molecular mechanisms of bone mechanical adaptation

Wolff’s law posits that bone adapts structurally to stress: growth occurs in regions of high load, while resorption predominates where stress is low (14). Frost’s “mechanostat” concept further emphasizes that bone senses mechanical cues and adjusts accordingly (15).

Piezo1, a mechanosensitive cation channel with a trimeric propeller-shaped structure, is expressed in tissues such as lung, kidney, bladder, vasculature, and bone (16–18). Its single transmembrane protein consists of 2,521 amino acids—the largest known transmembrane molecule—organized into 38 helices per subunit, forming peripheral blades that constitute the mechanosensing module. This structure is highly conserved across evolution (19).

Multiple studies identify Piezo1 as a core component of the skeletal mechanostat. Osteoblast-specific Piezo1 deficiency leads to bone loss, spontaneous fractures, and increased resorption, while conferring resistance to unloading-induced bone loss in mice (9). Mechanistically, Piezo1 influences type II and IX collagen expression through the YAP pathway: Activated YAP promotes the synthesis of type II and IX collagens, enhancing bone matrix integrity; meanwhile, collagens activate FAK signaling in osteoclasts via integrin αvβ3, inhibiting their excessive differentiation and ultimately maintaining the balance between bone resorption and formation. Deletion of Piezo1 in osteoblasts disrupts osteogenesis, causes skeletal fragility, and its expression declines with age in human OP patients (20). These findings firmly establish Piezo1 as a molecular bridge linking mechanical force to bone homeostasis.

At the functional level, Piezo1 maintains skeletal integrity via dual mechanisms: it upregulates osteoprotegerin (OPG) to inhibit osteoclastogenesis and simultaneously promotes osteoblast activity, particularly protecting against age-related cortical bone loss (21). Developmental studies show that Piezo1 deletion during embryogenesis (global knockout: Piezo1fl/fl; Sox2-Cre) induces bone deformities and fractures, whereas loss of function in adulthood (osteoblast-specific knockout: Piezo1fl/fl; OCN-Cre) directly causes osteoporosis (6). Together, evidence from both developmental biology and adult bone metabolism underscores Piezo1 as a central regulator of skeletal health. Its age-dependent expression provides a strong rationale for anti-osteoporosis therapies targeting Piezo1 activation.

## 2 Mechanical regulatory functions of Piezo1 and other mechanosensors in bone metabolism

### 2.1 Dominant role of Piezo1 in bone metabolism

Hindlimb suspension (HS) experiments show that unloading reduces bone strength in wild-type mice but not in Piezo1-knockout (KO) mice, suggesting that Piezo1 primarily regulates skeletal remodeling via osteoblasts (9). In osteocalcin (OCN)-specific KO mice, Piezo1 deletion caused shortened and weakened long bones, reduced bone mass, impaired osteoblast differentiation, and abolished mechanical loading–induced osteoblast–osteoclast coupling. By contrast, Piezo2 deletion had no significant impact on bone mass or bone length (22, 23). These results establish Piezo1 as the dominant mechanosensor in bone, with Piezo2 playing only a minor role.

**Figure 1:**
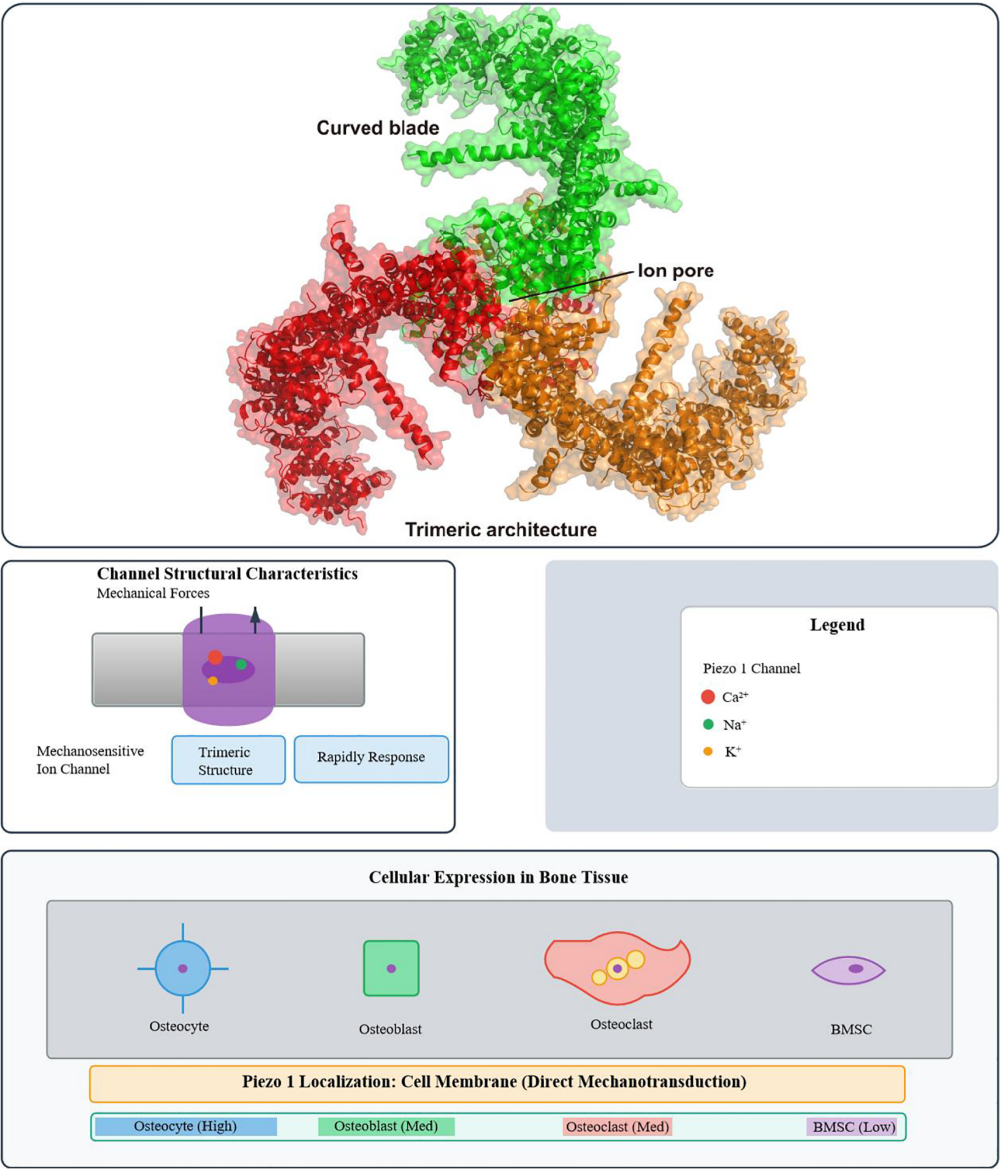
Structural characteristics of the Piezo1 ion channel and its distribution in bone tissue. (Top) Cryo-EM structure of Piezo1, highlighting its trimeric architecture, central ion pore, and curved blade domains. (Bottom) Schematic representation of Piezo1 expression in bone tissue. Piezo1 mediates rapid ion transport (Ca^2+^, Na^+^, K^+^) in response to mechanical force. It is highly expressed in osteocytes (the core mechanosensors), moderately in osteoblasts and osteoclasts, and weakly in bone marrow MSCs (BMSCs). Localized primarily at the cell membrane (direct mechanotransduction) and at low levels in the endoplasmic reticulum (involved in calcium homeostasis), Piezo1 converts mechanical cues into biochemical signals—with cell-specific functions (osteocytes, sensing fluid shear stress; BMSCs, regulating differentiation fate)—to regulate bone metabolism.

Piezo1 and Piezo2 form mechanosensitive cation channels (24), but Piezo1 is the primary transducer of membrane tension. Upon mechanical stimulation, Piezo1 opens to mediate Ca^2+^ influx, converting external forces into intracellular signals that drive mechanotransduction and cellular adaptation (25–27). This process is indispensable for bone-forming cell survival, differentiation, and matrix mineralization, as well as skeletal remodeling and regeneration (28) (**Figure 1**).To provide a comprehensive overview, **Figure 1** illustrates both the structural characteristics of Piezo1—highlighting its trimeric architecture, central ion pore, and curved blades—and its expression patterns in bone tissue, where it shows cell type–specific localization and functions.

Osteocyte apoptosis critically affects bone homeostasis. Excessive apoptosis, such as that induced by glucocorticoids (GCs), disrupts the lacunar–canalicular network, reducing fluid flow and connectivity, ultimately impairing bone quality (29, 30). This process involves caspase-3 activation and phosphorylation of proline-rich tyrosine kinase 2 (PYK2) and c-Jun N-terminal kinase (JNK). Conversely, mechanical stress promotes production of anti-apoptotic mediators (e.g., nitric oxide and prostaglandin E2), helping preserve osteocyte viability (31, 32).

In BMSCs, Piezo1 also mediates proliferation and osteogenic differentiation. *In vitro* cyclic mechanical stretch (CMS) increases proliferation and upregulates osteogenic markers (COL1A1, OSX, RUNX2) in rat BMSCs. Piezo1 knockdown significantly reduces these effects, underscoring its critical role in mechanotransduction (33). Osteocytes sense fluid shear forces via Piezo1, triggering Ca^2+^ signaling cascades that regulate bone remodeling (11). In osteoblasts and chondrocytes, Piezo1-mediated Ca^2+^ influx activates downstream ERK1/2 and PI3K/Akt pathways, promoting osteogenesis and regulating cartilage metabolism (34). In periodontal ligament cells, Piezo1 responds to orthodontic pressure, modulating alveolar bone remodeling (35, 36).

Bone mechanotransduction is a multi-level system. Osteocytes form a mechanosensing complex with dendritic networks, integrins, ion channels (e.g., ANO1), and primary cilia. Mechanical loading also induces osteocytes to release exosomes carrying regulatory miRNAs, potentially contributing to systemic homeostasis. In osteoblasts, Piezo1-mediated Ca^2+^ signaling interacts with ANO1 chloride channels to influence osteoclast regulation (37, 38). Moreover, bone microvascular endothelial cells participate in signal transmission, and unloading disrupts this function (39).

Wnt/β-catenin and RANKL signaling pathways are central to Piezo1-mediated mechanical regulation. Mechanical loading activates Wnt/β-catenin signaling, promoting osteoblast differentiation, while suppressing RANKL-mediated osteoclastogenesis (40). YAP/TAZ also function as mechanosensitive transcriptional regulators, guiding BMSC fate via Runx2. For example, loading enhances expression of Fgf23 and Mepe, genes critical for phosphate metabolism and bone mineralization (41).

### 2.2 Other mechanosensors in bone metabolism

#### 2.2.1 TRPV4 as a complementary mechanosensor

TRPV4 senses low-intensity, physiological mechanical deformation (0.1–1 dyne/cm², e.g., bone tissue hydrostatic pressure) and regulates chondrocyte differentiation, extracellular matrix metabolism, and osteogenic gene expression through Ca^2+^ influx. In contrast, Piezo1 responds to supraphysiological or injurious forces (≥5 dyne/cm², e.g., exercise-induced fluid impact). When Piezo1 is impaired (e.g., aging, knockout), TRPV4 partially compensates to sustain tissue homeostasis: mice with double knockout of Piezo1 and TRPV4 exhibit significantly more severe bone loss than Piezo1 single-knockout mice, with a 2.3-fold increase in osteoclast number (42, 43).

#### 2.2.2 GPR68 as a supplementary mechanosensor

GPR68 provides additional compensation by responding to mechanically associated environmental changes such as pH shifts and fluid shear stress, especially under inflammatory conditions. GPR68 activation reduces osteoclast-related factors via PLC–IP3 signaling. In osteoarthritis, upregulation of GPR68 suppresses aberrant cartilage degradation through Rap1A-dependent pathways, offering a non-Ca^2+^-dependent compensatory mechanism—under inflammatory conditions, this pathway can partially reverse the enhanced bone resorption caused by Piezo1 deficiency (44–46).

#### 2.2.3 Synergy among Piezo1, TRPV4, and GPR68

Piezo1 remains the central mechanotransducer, regulating bone remodeling, survival, and inflammatory responses (6, 47). TRPV4 and GPR68 act as compensatory systems: TRPV4 maintains Ca^2+^ signaling during physiological stimuli (0.1–1 dyne/cm²), while GPR68 compensates through pH-sensitive G-protein pathways. This redundancy across stimulus intensity and signaling modes ensures skeletal balance even when Piezo1 function declines.

## 3 Direct and systemic regulatory mechanisms of Piezo1 in osteoporosis

### 3.1 Direct regulation of bone cells by Piezo1

#### 3.1.1 Osteoblast differentiation

Under hydrostatic pressure, Piezo1 functions as a signaling hub that rapidly initiates osteogenic programs. Piezo1-mediated Ca^2+^ influx activates ERK1/2 phosphorylation cascades and promotes F-actin assembly—F-actin assembly further promotes the G1/S phase transition of osteoblasts by activating YAP nuclear translocation (upregulating Cyclin D1 expression) and enhances cell adhesion, providing cytoskeletal support for osteoblast proliferation. Agonists such as Yoda1 significantly enhance BMP2 expression, directing BMSCs toward osteogenesis while suppressing adipogenesis. Conversely, Piezo1 silencing reduces BMP2 expression and cell migration (48).

A newly identified agonist, MCB-22-174, activates the Piezo1/CaMKII/ERK axis, offering a therapeutic approach for disuse osteoporosis (49). Collectively, Piezo1 acts as a central conductor of osteogenic differentiation, orchestrating signaling pathways that coordinate bone formation.

#### 3.1.2 Cartilage differentiation and ossification balance

Piezo1 is highly expressed in chondrocytes, where it regulates responses to mechanical strain. Inhibition with GsMTx4 markedly diminishes chondrocyte mechanosensitivity (50). In inflammatory conditions, IL-1α enhances Piezo1 expression, causing Ca^2+^ overload and chondrocyte dedifferentiation, which predisposes to osteoarthritis (51).

During endochondral ossification, Piezo1 deletion disrupts key gene expression (e.g., Sox9, Col10a1), damaging growth plate structure and increasing fracture susceptibility (52). In osteoarthritis models, mechanical overload induces Piezo1-mediated Ca^2+^ influx that destabilizes the cytoskeleton and upregulates MMP13, accelerating cartilage degeneration (53). These findings underscore Piezo1 as a guardian of cartilage mechanohomeostasis, with dysfunction closely linked to degenerative joint disease.

### 3.2 Phenotypic differences of Piezo1 in skeletal development

#### 3.2.1 Developmental vs. adult bone homeostasis

During embryogenesis, Piezo1 is indispensable for skeletal development. Its deletion (Piezo1fl/fl; Sox2-Cre) causes cranial defects, cortical porosity, reduced strength, and aberrant STAT3 activation (54–56). In adults, Piezo1 inactivation (Piezo1fl/fl; OCN-Cre) results in cortical thinning, increased porosity, decreased trabecular bone volume, and reduced bone formation—hallmarks of high-turnover osteoporosis (57–59). These findings demonstrate stage-specific functions: Piezo1 orchestrates development early, and maintains homeostasis later in life.

#### 3.2.2 Aging and sex differences

Piezo1 expression declines with age, impairing osteoblast function and aggravating cortical bone loss. Activation of Piezo1 can reverse glucocorticoid-induced osteoporosis by restoring Wnt/β-catenin signaling (6). Genetic variants of Piezo1 are also linked to bone mineral density and fracture risk (60).

Estrogen deficiency further reduces Piezo1 expression, particularly in aging females: estrogen binds to the estrogen response element (ERE) in the Piezo1 promoter via ERα to promote its transcription; after estrogen deficiency, ERα-mediated transcriptional regulation of Piezo1 is lost, and simultaneous activation of the ROCK pathway leads to F-actin depolymerization, weakening cytoskeletal remodeling and reducing suppression of osteoclastogenesis (6, 61, 62). Estrogen deficiency also increases oxidative stress and reduces osteogenic activity, which synergize with Piezo1 loss. Mechanistically, Piezo1 deletion disrupts metabolism through the SIRT3–SDHA–OXPHOS axis, exacerbating impaired bone formation (63, 64). Furthermore, the Wnt/Ca^2+^ pathway, normally activated by Piezo1, is suppressed under estrogen deficiency, reducing osteogenesis (12, 36).

## 4 Indirect regulation of Piezo1 through non-bone cell networks

### 4.1 Vascular–immune axis: coordinated regulation of the bone microenvironment

In endothelial cells, Piezo1 functions as a mechanosensor that regulates vascular tone and blood flow distribution (65). Following radiation-induced bone injury, Piezo1 activation in bone marrow macrophages stimulates VEGFA release through the CaN/NFAT/HIF-1α pathway, thereby promoting vascular regeneration (66). Conversely, Piezo1 deletion downregulates PI3K–Akt and Notch signaling during fracture healing, impairing osteoblast maturation (67).

Under mechanical loading, periosteal myeloid cells differentiate into CD68^+^F4/80^+^ macrophages, which release thrombospondin-1 (TSP1) to activate TGF-β1 signaling, synergistically promoting bone formation (68). These findings indicate that Piezo1 regulates skeletal remodeling not only through direct mechanotransduction in bone cells but also by modulating the vascular–immune axis.

### 4.2 Gut–bone axis

Piezo1 also influences skeletal metabolism through the intestinal system. Intestine-specific Piezo1 deletion reduces serum serotonin (5-HT) levels—serotonin normally inhibits osteoblast proliferation—thus enhancing osteoblast activity and producing a high bone mass phenotype (69, 70). This finding identifies intestinal Piezo1 as a negative regulator of osteogenesis and highlights the gut–bone axis as an inter-organ regulatory network influencing skeletal health.

## 5 Core mechanosignaling pathways mediated by Piezo1

**Figure 2:**
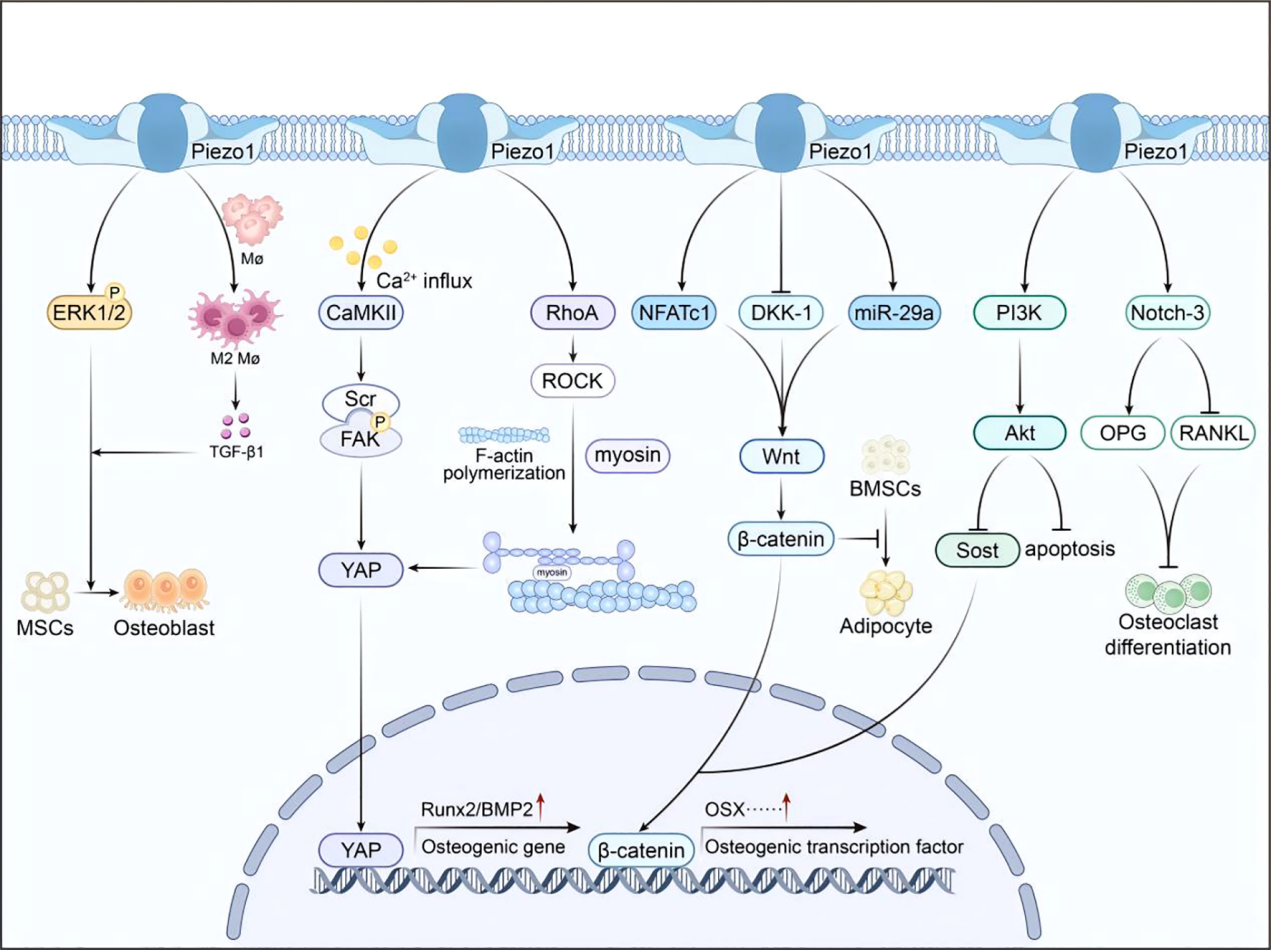
Integrated Piezo1 signaling network. Mechanical activation of Piezo1 triggers multiple downstream pathways (CaMKII–YAP, RhoA/ROCK, Wnt/β-catenin, ERK1/2, PI3K/Akt), collectively promoting osteogenesis and suppressing osteoclastogenesis.

Piezo1 integrates pathways into a unified mechanosignaling network that regulates osteogenesis and osteoclast activity (**Figure 2**). The following subsections detail the key components of this network.

### 5.1 CaMKII pathway: calcium signaling hub

Mechanical stimulation activates Piezo1, leading to Ca^2+^ influx and subsequent activation of Ca^2+^/calmodulin-dependent protein kinase II (CaMKII) (71). Activated CaMKII phosphorylates focal adhesion kinase (FAK) and Src—phosphorylated FAK/Src inhibits the activity of Hippo pathway kinase MST1/2, reducing YAP phosphorylation at Ser127 and thereby driving YAP nuclear translocation to regulate osteogenic gene expression (72). This pathway plays a critical role in pathological ossification, such as ankylosing spondylitis, where aberrant mechanical signaling promotes osteophyte formation. In osteoporosis, insufficient Piezo1 activation reduces CaMKII signaling, YAP nuclear localization, and osteogenic gene expression, resulting in impaired bone formation.

### 5.2 YAP/TAZ pathway: cytoskeletal remodeling switch

Piezo1-mediated mechanical stimulation activates the RhoA/ROCK pathway, inducing cytoskeletal remodeling through F-actin polymerization and myosin reorganization (47). This structural reorganization facilitates YAP nuclear translocation, which upregulates osteogenic transcription factors such as Runx2 and BMP2 (73). For example, triangular micropatterns enhance BMSC osteogenesis through this mechanism (74). In osteoporosis, weakened mechanical stimulation reduces Piezo1 activity, restricting cytoskeletal remodeling and YAP signaling, thereby impairing osteogenic differentiation and promoting adipogenesis.

### 5.3 Wnt/β-catenin pathway: bridge to bone metabolism

Piezo1 may activate the Wnt/β-catenin pathway via NFATc1 (75). Wnt activation drives β-catenin nuclear translocation, promoting transcription of osteogenic genes such as OSX while inhibiting adipogenesis (76). In osteoporosis, reduced Piezo1 activity attenuates Wnt/β-catenin signaling, leading to diminished osteogenesis and increased marrow adiposity.

### 5.4 ERK1/2 phosphorylation pathway: rapid response channel

Piezo1 activation by hydrostatic pressure or Yoda1 induces ERK1/2 phosphorylation, promoting BMSC osteogenic differentiation (77). Under physiological fluid shear stress, Piezo1 is upregulated in osteocytes, which activate Notch3 signaling to enhance OPG expression and suppress RANKL, thereby inhibiting osteoclastogenesis (11). Mechanical stretch also activates the PI3K/Akt pathway via Piezo1, downregulating Sost while enhancing Wnt/β-catenin signaling, thus driving osteogenesis (78). Moreover, Piezo1 activation reverses dexamethasone-induced osteocyte apoptosis through PI3K/Akt-mediated Ca^2+^ signaling (79, 80).

### 5.5 Multi-pathway synergy under mechanical stimulation

**Figure 3:**
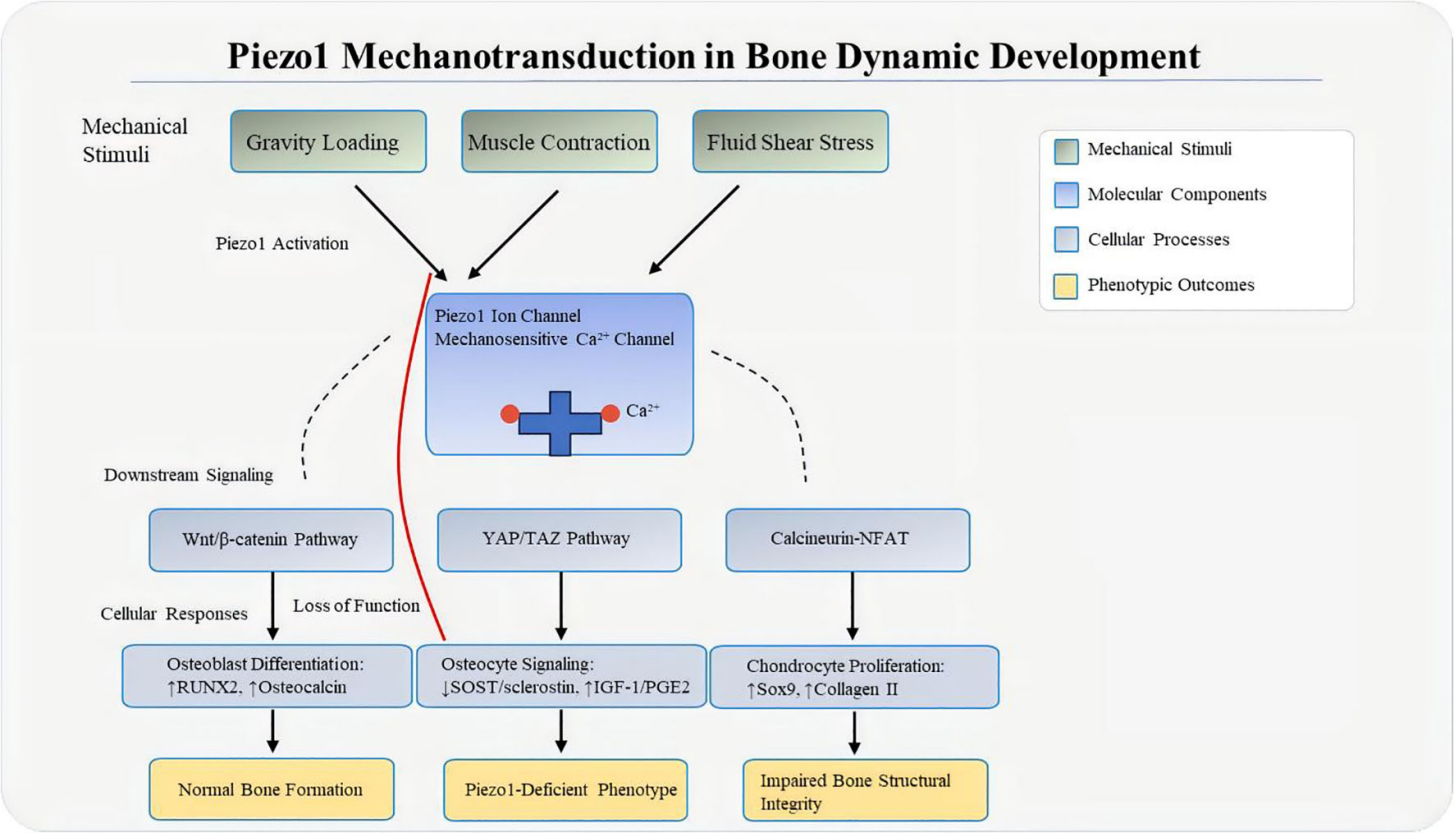
Mechanical loading–Piezo1 axis. Mechanical forces such as fluid shear stress, compression, and matrix stiffness deform the bone matrix, increasing membrane tension and triggering Piezo1 activation—with force-specific cellular targets (gravitational loading: osteoblasts; muscle contraction: osteocytes; FSS: osteocytes + vascular endothelial cells). Piezo1 then mediates Ca^2+^ influx, which activates downstream mechanosignaling cascades (YAP/TAZ, Calcineurin-NFAT, Wnt/β-catenin), converting physical loading into biochemical responses that regulate bone homeostasis. In the “Loss of Function” state, molecular changes include reduced YAP nuclear translocation, decreased Wnt3a expression, and increased RANKL/OPG ratio, leading to impaired bone structural integrity.

Piezo1 integrates multiple pathways during mechanical interventions. For instance, piezoelectric microvibration (PMVS) activates Wnt/β-catenin signaling by upregulating miR-29a and suppressing DKK-1 (81, 82). Additionally, Piezo1 polarizes macrophages toward the M2 phenotype: Piezo1-mediated Ca^2+^ influx activates the STAT6 pathway, upregulating M2 markers (Arg1, IL-10) and promoting TGF-β1 precursor maturation, thereby stimulating TGF-β1 secretion to enhance BMSC osteogenesis (83). This coordinated multi-pathway network ensures precise bone responses to mechanical stimuli. In osteoporosis, disruption of this network leads to impaired bone remodeling (**Figure 3**).

## 6 Interactions of Piezo1 with different pathways in specific bone cell types

### 6.1 Interaction with the CaMKII pathway

In adipose-derived stem cells (ADSCs), Piezo1-mediated Ca^2+^ influx activates CaMKII phosphorylation, enhancing β-catenin transcriptional activity and nuclear translocation, ultimately promoting osteogenesis (84). In osteoblasts and related cells, Piezo1 activation under stress also triggers CaMKII signaling, which synergizes with the Wnt/β-catenin pathway to regulate osteogenic differentiation (71).

### 6.2 Interaction with the YAP/TAZ pathway

In human dental follicle cells (hDFCs), cyclic tensile stress activates Piezo1, inducing Ca^2+^ influx that promotes YAP nuclear translocation and upregulates osteogenic genes (85). In BMSCs, Piezo1 integrates with YAP signaling, regulating target genes such as ATF4 via β-catenin and influencing proliferation and osteogenesis (7, 86). In valvular interstitial cells (VICs), Piezo1 activation drives Ca^2+^-dependent YAP signaling, enhancing osteogenesis through GLS1-mediated glutamine metabolism (87). In osteoblasts and osteosarcoma cells, Piezo1-mediated Ca^2+^ influx is essential for YAP/TAZ activation, which regulates cell motility and bone-associated processes (88).

### 6.3 Interaction with the Wnt/β-catenin pathway

In periodontal ligament cells (PDLCs), compressive force upregulates Piezo1 and β-catenin, while Piezo1 inhibition decreases β-catenin activity and osteogenic differentiation, modulating alveolar bone remodeling (35). In hDFCs, Piezo1 activation (e.g., Yoda1) enhances Wnt3a and β-catenin expression, activating canonical osteogenesis (89). In BMSCs and osteoblasts, Piezo1 promotes β-catenin nuclear translocation via Ca^2+^ influx, cooperating with CaMKII to support osteogenesis. Importantly, Piezo1 restores suppressed Wnt/β-catenin activity under microgravity, mitigating bone loss (75). In ADSCs, compressive stress–induced Piezo1 activation enhances β-catenin transcriptional activity and contributes to bone remodeling (36).

### 6.4 Interaction with the ERK1/2 pathway

In BMSCs, Piezo1 activation by hydrostatic pressure or Yoda1 triggers Ca^2+^ influx and ERK1/2 phosphorylation, promoting osteogenesis; this effect is abolished under Ca^2+^ deficiency (49, 90). In osteoblasts and osteosarcoma cells, Piezo1-mediated Ca^2+^ entry activates ERK via the MAPK cascade, which also cross-talks with YAP and Wnt/β-catenin signaling (91). In periodontal ligament cells and chondrocytes, Piezo1 activation engages ERK1/2 via PI3K–Akt/NF-κB, indirectly regulating proliferation and bone-related processes (92–94).

## 7 Therapeutic implications

**Figure 4:**
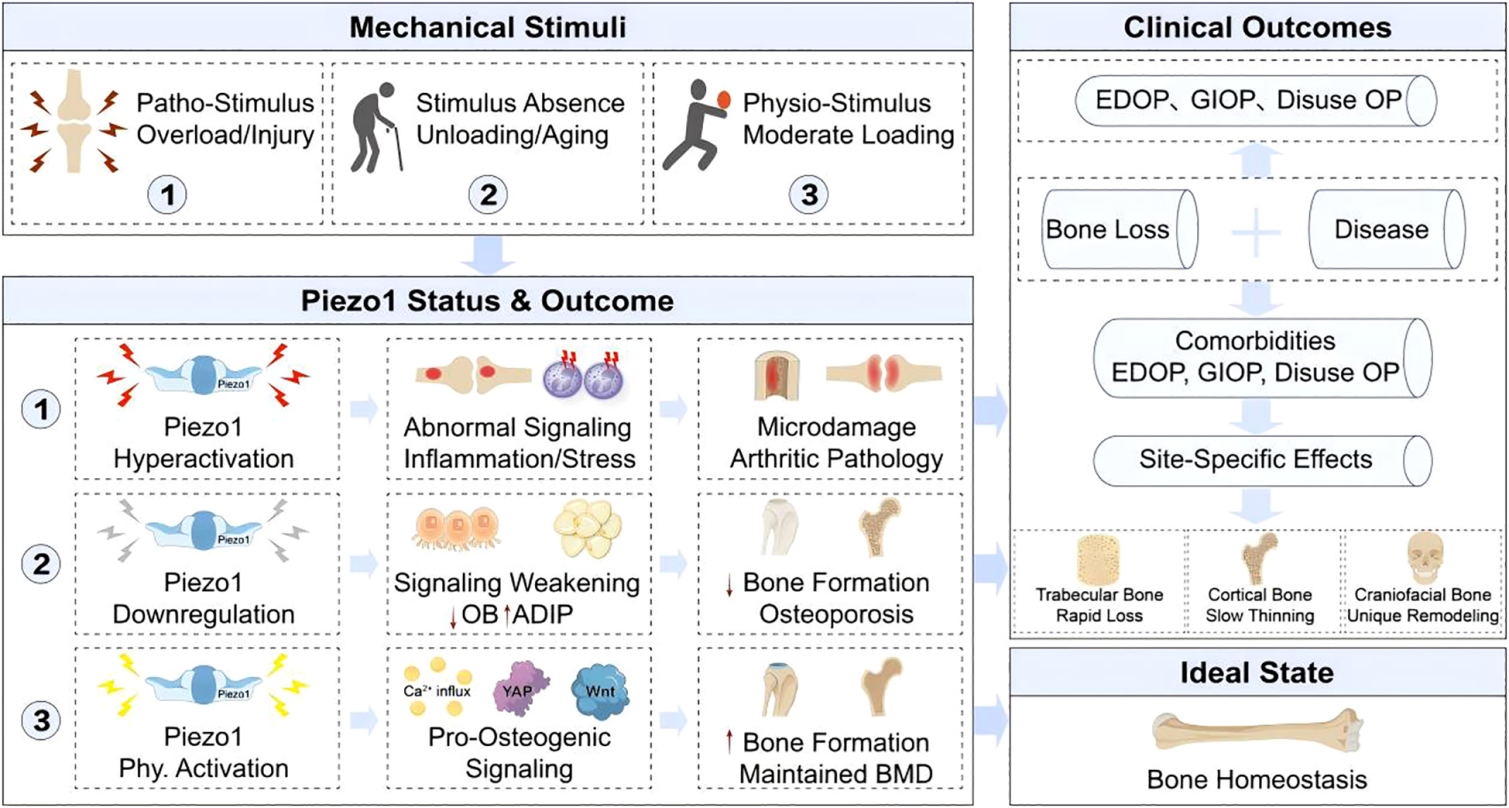
Pathophysiological outcomes of Piezo1 dysregulation and therapeutic implications. The spectrum of mechanical loading determines Piezo1 activity states, which govern skeletal fate. Physiological stimuli maintain Piezo1 activation and pro-osteogenic signaling, ensuring homeostasis. Loss of loading (e.g., aging, disuse) causes Piezo1 downregulation, shifting MSC fate toward adipogenesis and leading to bone loss (e.g., EDOP, GIOP). Conversely, pathological overloading hyperactivates Piezo1, inducing aberrant signaling and inflammation that contribute to arthritic pathology. This framework highlights key modifiers, including sex hormones (estrogen deficiency), bone site-specific responses (craniofacial vs. long bone remodeling), and comorbidities, underscoring the therapeutic goal of achieving precise Piezo1 modulation.

Building upon the detailed molecular mechanisms of Piezo1 signaling, we propose a comprehensive pathophysiological framework that links mechanical input to skeletal outcomes (**Figure 4**). This model delineates how the spectrum of mechanical loading—from physiological stimulation to overload or absence of force—dictates Piezo1 activation states, thereby governing the fate of bone cells and ultimately determining bone mass and quality. Crucially, this framework incorporates critical modifiers of bone homeostasis, including hormonal status (e.g., estrogen deficiency), bone site-specific remodeling patterns, and common comorbidities.

This mechano-dependent duality is exemplified in the context-specific, bidirectional regulation of osteoclasts by Piezo1. For example, in peri-prosthetic models, fluid shear stress mediated by Piezo1 exhibits frequency-dependent effects: low-frequency, high-amplitude stimulation enhances resorption, whereas high-frequency, low-amplitude stimulation promotes bone formation (95). In orthodontic models, Piezo1 activation increases the RANKL/OPG ratio, elevating TRAP^+^ osteoclast numbers (96). Conversely, prolonged high-amplitude fluid shear stress induces osteoclast formation by inhibiting the sarcoplasmic reticulum Ca^2+^ pump (97).

Piezo1 can also suppress osteoclastogenesis by upregulating OPG; its deficiency increases endocortical resorption (6). Thus, selective Piezo1 agonists (e.g., Yoda1 and its optimized derivatives) represent promising candidates for anti-osteoporosis therapies (6). In fracture repair, Piezo1 regulates endochondral ossification by modulating HIF-1α signaling in chondrocytes (98).

### 7.1 Broad regulatory effects

Engineered biomaterials exploit Piezo1 signaling to enhance bone repair. For example, oleic acid–modified iron oxide nanoparticles (IO-OA/PLGA) increase Piezo1 expression under magnetic fields: magnetic fields induce local mechanical vibration (10–50 Hz) of IO-OA/PLGA particles, activating Piezo1 channels via membrane tension, while particles slowly release oleic acid to promote Piezo1 transcription (99). Similarly, 3D-printed Ti2448 alloy scaffolds enhance angiogenesis and osteogenesis via Piezo1/YAP signaling, while titanium dioxide nanotubes stimulate Piezo1-mediated osteogenesis (100).

In fracture healing, reduced Piezo1 expression delays callus mineralization, whereas Yoda1 treatment increases BV/TV and bone mineral density, accelerating cartilage and callus maturation (101). Mechanical interventions such as low-intensity pulsed ultrasound or piezoelectric microvibration (PMVS) also activate Piezo1, promoting osteoblast precursor proliferation and migration, thereby improving bone strength (48).

### 7.2 Exercise therapy and rehabilitation

Exercise activates Piezo1 through cyclic loading, enhancing Ca^2+^ influx and Akt phosphorylation, which promote osteogenesis and skeletal muscle protein synthesis (102–104). Radial extracorporeal shock wave (R-ESW) therapy stimulates Piezo1/CaMKII/CREB signaling in senile osteoporosis (SOP) patient-derived BMSCs, enhancing their osteogenic and angiogenic capacity while reducing bone loss in animal models (105). These findings highlight Piezo1 as a therapeutic target linking musculoskeletal rehabilitation and osteoporosis treatment (**Tables 1**, **2**).

**Table 1:** Evidence summary of exercise and physical interventions targeting *Piezo1* for osteoporosis rehabilitation.

Intervention method	Study population	Mechanism/effect	Reference
Whole-body Vibration Training (WBV)	Postmenopausal women with osteoporosis	Increase bone mineral density, improve muscle strength and balance ability	(106)
Pulsed Electromagnetic Field (PEMF)	Rat model of hindlimb unloading osteoporosis	Inhibit osteoclastogenesis and reduce bone loss	(107)
Weight-bearing Training	Elderly population (≥65 years old)	Stimulate bone formation through mechanical loading	(108, 109)
Resistance Training	Patients with osteoporosis	Enhance muscle strength and improve bone metabolic indicators	(110)
Multicomponent Training Program	Patients with osteoporosis-related sarcopenia	Integrate multiple exercise modes to synergistically improve the musculoskeletal system	(111)
High-intensity Impact Training	Postmenopausal women	Stimulate bone remodeling through high mechanical stress	(112)
Proprioceptive Training	Elderly patients with osteoporosis	Improve body balance ability and reduce the risk of falls	(108)
Electrical Stimulation Therapy	Disuse osteoporosis model	Substitute for mechanical loading to stimulate bone formation	(113)
Vibration Combined with Exercise Intervention	Patients with osteoporotic fractures	Synergistically enhance bone density and muscle function	(114)
Mind-body Comprehensive Training (Tai Chi, Yoga, etc.)	Elderly patients with osteoporosis	Improve body control ability through neuromuscular coordination	(115)
Progressive Resistance Training	Patients with osteoporosis complicated with sarcopenia	Synchronously improve bone density and muscle mass	(111)
Aquatic Exercise Therapy	Patients in the rehabilitation period of osteoporosis	Safely enhance bone loading in a weight-bearing-reduced environment	(116)

**Table 2:** Piezo1 Therapeutic strategies.

Intervention	Mechanism	Efficacy	Safety	Phase	Reference
Yoda1 (classic agonist)	Binds Piezo1, opens channel, activates Ca^2+^ signaling	Synergistic with vibration; reverses dexamethasone-induced bone loss	Long-term use may cause cortical perforation; low-dose safe	Research tool/Preclinical	(23)
MCB-22-174 (novel agonist)	Activates Piezo1 → Ca^2+^ → ERK/CaMKII	↑ Bone volume 36–43%; ↑ Strength 35%	Good cardiovascular profile; no organ toxicity	Preclinical stage	(33)
(Thiadiazol-2-yl) pyrazine derivatives	Piezo1 agonist (EC_50_ 2.2 μM), activates Ca^2+^→ERK	Reduces bone loss by 27% in rats	Good safety margin in animals	Preclinical stage	(117)
SJTU BXA series compounds	Novel bone-targeted Piezo1 agonists (details undisclosed)	Better oral exposure vs Yoda1	GLP tox studies ongoing	Preclinical stage	(118)
HIU (High-intensity ultrasound)	Stimulates Piezo1-Ca^2+^-ERK1/2 axis	Accelerates fracture healing (+33%); ↑ Bone density	No soft tissue injury; long-term safety TBD	Technology translation stage	(12)
rESW (Radial extracorporeal shockwave)	Activates Piezo1 → Ca^2+^ → CaMKII/CREB	Promotes BMSC osteogenesis & angiogenesis; ↑ Bone density +25%	CE/FDA approved device; good safety record	Indication expansion research	(105)
LMV/PMVS (Low-magnitude vibration)	Activates Piezo1 → miR-29a → Wnt3a	Restores BMD+18%; ↓ CTX-1	No adverse effects; parameters safe	Near clinical application	(119)
Yoda1-loaded bone-targeted nanocarrier	Nanocarriers deliver Yoda1 to bone, sustain Piezo1 activation	Effective synchronous bone–vascular repair in rats	Reduced systemic toxicity; no major organ damage	Proof-of-concept stage	(120)
Peptide regulators	Lipidated peptides modulate Piezo channels (patent)	No osteoporosis data yet	Safety data unavailable	Early exploratory stage	(33)

## 8 Difficulties and challenges

Despite significant progress, research on Piezo1 faces several challenges that hinder its translation into clinical therapies. A major obstacle is the precise modulation of mechanical stimulation parameters. Mechanical loading exerts bidirectional effects: low-frequency, high-amplitude stress tends to promote osteoclast-mediated resorption, whereas high-frequency, low-amplitude stress favors osteogenesis. Current studies on vibration frequency focus on 20–100 Hz, and ultrasound intensity on 0.5–2 W/cm², but optimal parameters vary significantly among populations of different ages (young vs. elderly) and genders (male vs. postmenopausal female), and an individualized parameter database is lacking—determining optimal parameters to maximize the osteogenic benefits of Piezo1 activation remains unresolved (60).

Another challenge lies in the complexity of signaling crosstalk. Piezo1 activates multiple downstream pathways, including PI3K/Akt, ERK, and YAP, through Ca^2+^ influx. The relative contribution and interaction of these cascades in osteocytes, BMSCs, and osteoclasts are still poorly defined. Moreover, aging and inflammation further complicate regulation: declining Piezo1 expression reduces Wnt/β-catenin signaling, while pro-inflammatory cytokines such as IL-1α can cause Piezo1 overactivation, leading to Ca^2+^ overload and chondrocyte apoptosis (51). Dissecting the molecular switches that govern this imbalance is critical for targeted intervention.

Piezo1 also interacts with other mechanosensing systems. Evidence suggests potential cross-talk with focal adhesion complexes and proteins such as Kindlin-2, as well as with other ion channels like connexin 43 hemichannels, but the specific molecular cascades remain largely unknown. In addition, Piezo1 plays a role in coordinating systemic regulatory axes, including the vascular–immune–bone and gut–bone axes. However, their dynamic integration is incompletely understood. For example, Piezo1 regulates macrophage polarization, VEGFA release, and vascular regeneration, yet how these processes interact during bone repair remains unclear. Similarly, intestine-specific deletion enhances osteogenesis by reducing circulating serotonin, which paradoxically contrasts with Piezo1’s direct pro-osteogenic role in bone tissue—single-cell sequencing can be used to analyze differences in downstream target genes of Piezo1 between intestinal epithelial cells and osteocytes, clarifying the regulatory hierarchy of serotonin-dependent and independent pathways (69, 70). Clarifying the hierarchy of such inter-organ signals is an urgent research priority.

Finally, pharmacological limitations represent a major bottleneck. Current Piezo1 agonists, such as Yoda1, have been tested primarily in animal models, but specific bone-targeted delivery systems are lacking. Given Piezo1’s broad expression across multiple tissues, concerns about off-target effects remain significant. Furthermore, the regulatory mechanisms controlling Piezo1’s dynamic expression during aging and disease progression are poorly characterized, complicating the selection of therapeutic timing (7).

## 9 Summary and perspectives

Piezo1 has emerged as the central mechanotransducer in bone tissue, converting external mechanical stimuli such as fluid shear stress and mechanical stretch into Ca^2+^ influx and activating downstream signaling pathways including PI3K/Akt, ERK, YAP/TAZ, and Wnt/β-catenin. Through these mechanisms, Piezo1 orchestrates the balance between osteoblast and osteoclast activity, regulates BMSC differentiation, and coordinates vascular–immune interactions, thereby playing a pivotal role in the pathogenesis and progression of osteoporosis. Declining Piezo1 expression with age or under pathological conditions such as estrogen deficiency directly impairs skeletal mechanoresponsiveness, contributing to reduced osteogenesis, increased bone resorption, and ultimately bone fragility.

Therapeutically, Piezo1 offers a promising target for intervention. Agonists such as Yoda1 and MCB-22–174 restore mechanotransduction and ameliorate bone loss in disuse, glucocorticoid-induced, and aging-related osteoporosis models. Mechanical therapies—including exercise, vibration, ultrasound, and shock wave treatment—also act through Piezo1 to promote osteogenesis, providing a theoretical basis for rehabilitation strategies. Furthermore, Piezo1’s systemic roles extend beyond bone tissue. In the gut–bone axis, intestine-specific Piezo1 deletion reduces circulating serotonin, indirectly enhancing osteoblast proliferation, while in the vascular–immune axis, Piezo1 regulates macrophage polarization and angiogenic factor release, contributing to bone repair. These findings suggest that Piezo1 functions not only as a local mechanosensor but also as a systemic regulator of skeletal homeostasis.

Future research should focus on several key directions. First, the development of selective Piezo1 agonists with optimized pharmacokinetic properties and bone-targeted delivery systems—e.g., modifying nanocarriers with bisphosphonates (high affinity for bone hydroxyapatite) or designing pH-sensitive carriers (bone microenvironment pH ≈ 5.5)—is crucial to reduce off-target risks (120). Second, building a database of individualized mechanical parameters based on genotype, age, and hormonal status could enable precision therapies using mechanical interventions or Piezo1 modulators. Third, advanced tools such as single-cell sequencing and *in vivo* Ca^2+^ imaging are needed to map Piezo1’s spatiotemporal activation patterns and to clarify its crosstalk with other key signaling pathways. Fourth, resolving the paradox between intestinal Piezo1 and bone Piezo1, as well as delineating the hierarchy of inter-organ regulatory networks, will be essential for fully understanding its systemic roles. Finally, the integration of Piezo1 agonists with established osteoporosis drugs, such as bisphosphonates—their combination may synergistically enhance BMD via Wnt/β-catenin (agonists: promote osteogenesis; bisphosphonates: inhibit resorption), with caution for Piezo1 overactivation-induced Ca^2+^ overload (51)—and the development of wearable mechanosensing devices for real-time feedback may provide novel strategies for long-term management and personalized rehabilitation (6, 121).

In summary, Piezo1 represents a pivotal molecular hub at the interface of biomechanics and bone biology. By bridging mechanical loading, cellular signaling, and systemic regulation, it not only provides new insights into the pathogenesis of osteoporosis but also opens avenues for innovative therapeutic strategies that combine pharmacological, mechanical, and bioengineering approaches.
